# The Causality Inference of Public Interest in Restaurants and Bars on Daily COVID-19 Cases in the United States: Google Trends Analysis

**DOI:** 10.2196/22880

**Published:** 2021-04-06

**Authors:** Milad Asgari Mehrabadi, Nikil Dutt, Amir M Rahmani

**Affiliations:** 1 Department of Electrical Engineering and Computer Science University of California Irvine Irvine, CA United States; 2 Department of Computer Science University of California Irvine Irvine, CA United States; 3 Department of Cognitive Sciences University of California Irvine Irvine, CA United States; 4 School of Nursing University of California Irvine Irvine, CA United States; 5 Institute for Future Health University of California Irvine Irvine, CA United States

**Keywords:** bars, coronavirus, COVID-19, deep learning, infodemiology, infoveillance, Google Trends, LSTM, machine learning, restaurants

## Abstract

**Background:**

The COVID-19 pandemic has affected virtually every region in the world. At the time of this study, the number of daily new cases in the United States was greater than that in any other country, and the trend was increasing in most states. Google Trends provides data regarding public interest in various topics during different periods. Analyzing these trends using data mining methods may provide useful insights and observations regarding the COVID-19 outbreak.

**Objective:**

The objective of this study is to consider the predictive ability of different search terms not directly related to COVID-19 with regard to the increase of daily cases in the United States. In particular, we are concerned with searches related to dine-in restaurants and bars. Data were obtained from the Google Trends application programming interface and the COVID-19 Tracking Project.

**Methods:**

To test the causation of one time series on another, we used the Granger causality test. We considered the causation of two different search query trends related to dine-in restaurants and bars on daily positive cases in the US states and territories with the 10 highest and 10 lowest numbers of daily new cases of COVID-19. In addition, we used Pearson correlations to measure the linear relationships between different trends.

**Results:**

Our results showed that for states and territories with higher numbers of daily cases, the historical trends in search queries related to bars and restaurants, which mainly occurred after reopening, significantly affected the number of daily new cases on average. California, for example, showed the most searches for restaurants on June 7, 2020; this affected the number of new cases within two weeks after the peak, with a *P* value of .004 for the Granger causality test.

**Conclusions:**

Although a limited number of search queries were considered, Google search trends for restaurants and bars showed a significant effect on daily new cases in US states and territories with higher numbers of daily new cases. We showed that these influential search trends can be used to provide additional information for prediction tasks regarding new cases in each region. These predictions can help health care leaders manage and control the impact of the COVID-19 outbreak on society and prepare for its outcomes.

## Introduction

The entire world is currently being significantly affected by a global virus pandemic. The first case of this virus, SARS-CoV-2, was reported in China in December 2019, and the first case outside China was discovered in January 2020 [1]. In February, the World Health Organization named the disease caused by this virus COVID-19 [2].

Worldwide, as of July 19, 2020, there had been approximately 14,400,000 confirmed cases of COVID-19, with 604,000 deaths [3]. The United States of America, with 3,830,000 confirmed cases and 143,000 deaths, was the most affected country in the world. In some states, such as California, the numbers are still increasing, while in some other states, such as New York, the peak has passed and the average number of daily new cases is decreasing.

Due to the rapid spread of SARS-CoV-2, finding effective reasons for its spread can play a significant role in prevention policies. Using data mining and time series analysis methods, it is possible to investigate the impact of different phenomena on time series data. For example, in economics, different studies have modeled the temporal relationships of two or more time series (eg, the relationship between oil and gold prices) using these methods [4]. Wang et al [5] used the same causality inference methods to determine whether a relationship exists between the main air pollutants and the mortality rate of respiratory diseases.

Through the study of infodemiology, which was first introduced by Eysenbach [6], it is now possible to extract knowledge from real-time and inexpensive data from web-based sources. These sources reflect the status of public health and answer the question of “what people are doing [7].” Conventionally, the collection of such information has been based on data collected by public health agencies and personnel [8]. However, it is now possible to extract global health information using web-based data mining [9]. Google search trends, for instance, can be a useful tool for reflecting public interests and concerns during different periods [10-12]. Morsy et al [13] considered the searches related to Zika virus to predict confirmed cases in Brazil. During the COVID-19 outbreak, different studies have investigated the correlation of web-based data and cases of SARS-CoV-2. Kutlu et al [14] investigated the correlation of dermatological diseases obtained by specific Google search trends with the COVID-19 outbreak. In addition, Google Trends has been used to predict and monitor COVID-19 cases worldwide [10,15-20]. Multiple studies have involved analysis of data related to the United States to correlate search trends and COVID-19 cases [21-26]. Although these studies consider the predictive ability of search trends on future confirmed cases, their search queries were limited to the symptoms and keywords related to the virus. For example, Ayyoubzadeh et al [10] investigated concepts related to COVID-19, such as hand washing, hand sanitizer, and antiseptic, as input features to predict the incidence of COVID-19 in Iran. However, these studies only considered the correlation of search trends with the spread of SARS-CoV-2, and no causality analysis has been performed.

In this paper, we were interested to investigate the effect of the reopening of in-store shopping on COVID-19 cases rather than searches directly related to the virus. Therefore, we considered the causality effect and predictive ability of search terms related to bars and restaurants on the number of daily new cases in different US states and territories. We analyzed the states and territories with the highest and lowest numbers of daily new cases to investigate the effect of Google searches with higher confidence.

In addition to linear correlation analysis between the search trends and COVID-19 cases, we used statistical causality methods to investigate the influential confidence of these methods on daily new COVID-19 cases.

## Methods

### Data Sets

For our analysis, we obtained the numbers of daily cases of COVID-19 in the United States using the COVID Tracking Project [27], which is publicly available. This project compiles daily statistics, including the numbers of positive and negative tests, hospitalization, available ventilators, and the number of deaths, in each US state and territory. For this study, we considered the data from a period of approximately three months, from April 9 to July 7, 2020, which contains 5040 data points for 56 states and territories.

For infodemiology studies, multiple sources can provide information regarding health informatics. Twitter and Google Trends are among the most popular data sources that have been used to track outbreaks [18]. Although in some studies, social media posts (eg, Twitter) have been leveraged for time series forecasting (eg, the stock market [28]), in this research, we selected Google Trends for the following reasons. First, for our analysis, we required access to location (ie, state) information; however, location is not available by default in social media platforms. More precisely, social media users must opt in to the use of location features (eg, tweeting with location), which limits the amount of available data. Second, search engines (eg, Google Trends) represent a wider scope of participants (eg, age, ethnicity, socioeconomic status) and are more universal than social media platforms (eg, Twitter) requiring memberships. In other words, Google Trends is a better proxy for the entire population in this case [29]. Lastly, social media is often used for idea and news sharing, whereas search engines are more informative with respect to searches for venues such as bars and restaurants.

For these reasons, we decided to use Google Trends to determine the public interest in bars and restaurants with daily resolution. We followed the methodology presented in [30] to obtain the results. We used queries for each state or territory from April 9 to July 7, 2020, for 45 available states and territories in the Google Trends application programming interface. For restaurants and bars, we chose *dine-in restaurants that are open near me* and *bars near me* as our queries, respectively. Throughout the remainder of this paper, we refer to “bar searches” and “restaurant searches” as the Google Trends data for the queries used to retrieve data related to bars and dine-in restaurants, respectively.

We did not narrow the category, as the keywords were specific [30]. Google Trends does not provide the number of queries per day. Instead, it provides a normalized number between 0 and 100, where 0 refers to a low volume of data for the query while 100 refers to the highest popularity for the query [31]. To be consistent with Google Trends values, we normalized the number of daily new cases in the United States between 0 and 100 in our analysis.

Aggregating data from the Google Trends results and COVID-19 daily cases and removing missing values resulted in available data for 45 US states and territories. Although all the results for all the states and territories are provided in Multimedia Appendices 1-4, we categorized our analysis into two different groups. The first group included the 10 states or territories with the highest numbers of daily new cases as of July 7, 2020, which consisted of Texas, Florida, California, Arizona, Georgia, Louisiana, Tennessee, North Carolina, Washington, and Pennsylvania. The second group included the 10 states or territories with the lowest numbers of daily new cases as of July 7, 2020: Kansas, Hawaii, New Hampshire, Maine, West Virginia, Rhode Island, Connecticut, Montana, Nebraska, and Delaware.

All the data used in this study are publicly available and are therefore exempted from the requirements of the Federal Policy for the Protection of Human Subjects under Category 4.

### Statistical Analysis

#### Correlation and Causation

To analyze the linear correlation of two time series, the Pearson correlation was used. The value of this correlation ranges from –1 to 1; these values show negative and positive correlations, respectively. Our analysis measured the Pearson correlation between the trends of search queries (ie, restaurants and bars) and the daily new cases of COVID-19 in each state.

In addition, we used Granger causality [32] to model the influence of past values of a time series on new values of another time series. Cross-correlation (lag correlation) is not an appropriate method in this context because due to its symmetrical measurement, it does not explain the causation. However, Granger causality tests whether the past values of a time series X cause the current values of another time series Y. Hence, in this study, the null hypothesis is that the past values of X do not affect the current values of Y. If the *P* value is less than the marginal value (.05), we can reject the null hypothesis. In our analysis, we reported *P* values for the influence of each aforementioned search query on the number of daily new cases. One of the main assumptions of modeling the influence of time series on each other is their stationarity. To test this characteristic, we used the augmented Dickey-Fuller (ADF) test [33] as our unit root test (Multimedia Appendix 4). This test determines the effect of a trend in the creation of the time series. In other words, it determines how strongly a trend defines a time series. The alternative hypothesis in the ADF test is the stationarity of the time series. 

In this study, because the time series were not stationary, we applied first differencing on search trends and second differencing on daily new cases to ensure that all three series were stationary. For the statistical analysis, we used the Python statsmodels package [34].

#### Vector Autoregression

In our study, we leveraged the fact that search trends may impact the number of daily new cases in the future; hence, a vector autoregression (VAR) [35] model for each region was fitted to the data. A VAR model takes into account the influence of the past values of time series X and Y on the current values of time series Y with a given lag order. The lag order with the lowest Akaike information criterion was chosen in this study. Because symptoms may appear within 2-14 days after exposure to SARS-CoV-2 [36], a maximum of 14 lags was used. The equation for the VAR model with two lags is summarized below:

*Y_t_* = *α* + *β*_1_*X_t_*_–1_ + *β'*_1_*X_t_*_–2_ + *β'*_2_*Y_t_*_–1_ + *β'*_2_*X_t_*_–2_ + *∈_t_* **(1)**

In equation 1, *Y_t_* represents the value of time series *Y* at time *t*, which consists of a combination of previous lag values from Y and X with different weights *β*, *β'* and random white noise, *∈_t_*. In other words, this equation models the importance of past values of the considered time series, as well as a secondary time series, for the estimation of the current value. We fitted a VAR model with different lag orders to perform the Granger causality test. Although the VAR model was used to compute the Granger causality, we did not use this model for the prediction task. Instead, we used a deep learning architecture for our prediction task.

#### Long Short-Term Memory

A long short-term memory (LSTM) [37] model is a type of recurrent neural network that is useful for time series prediction. LSTM models capture the long-term effect of a time series as well as its most recent values. In this study, we used LSTMs to predict the daily new cases using two sets of features: (1) the historical values of the new cases time series and (2) additional information from the search query time series. We used 70% of the data for training, and the remaining data were used for evaluation of the model. Root mean square error (RMSE) was selected as the performance metric. RMSE can be calculated as follows:

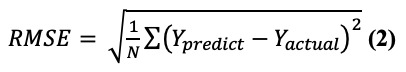



In equation 2, *N* is the number of samples, *Y_predict_* is the predicted value, and *Y_actual_* is the actual value of the time series.

We calculated RMSEs for three models: (1) the baseline model, which uses only the past values of the new cases time series for the prediction, (2) the model that uses the past values of restaurant searches along with the past values of the new cases time series, and (3) the model that combines the information from the time series of daily cases and the bar searches.

**Figure 1:**
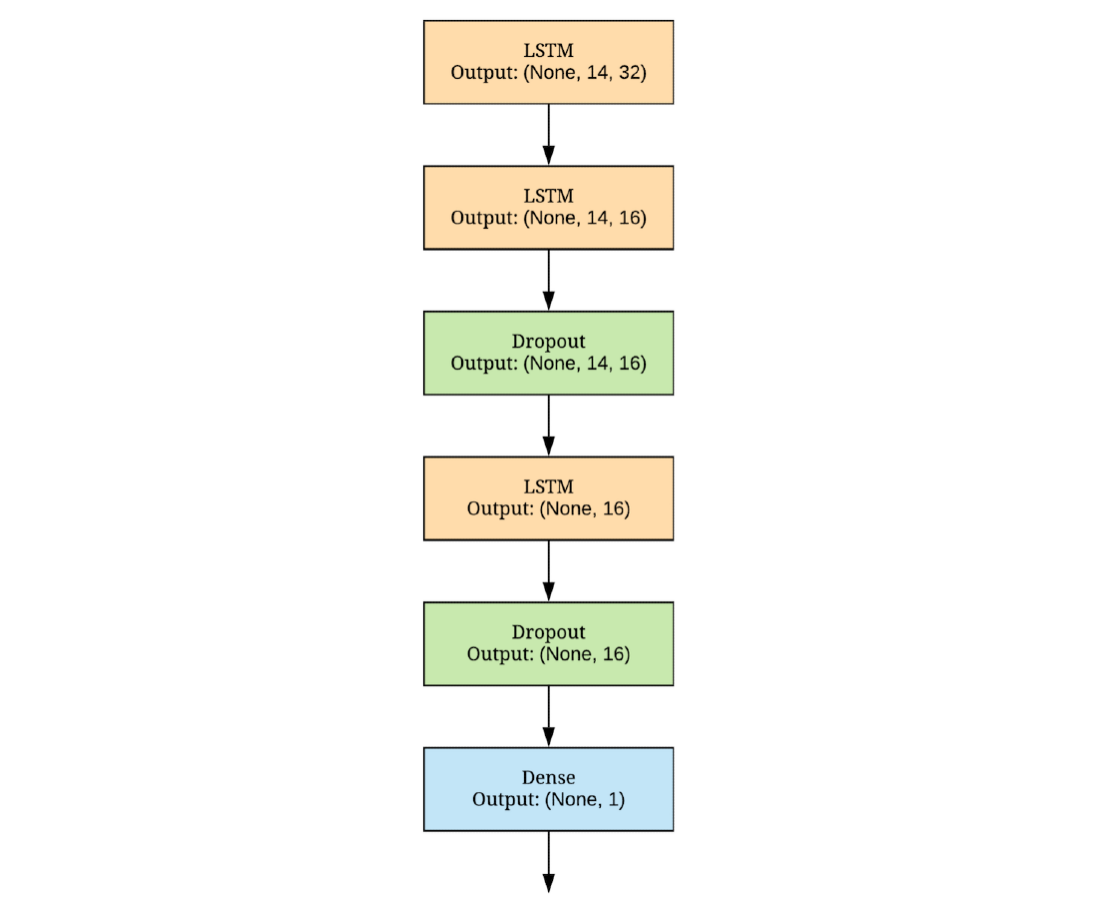
The proposed model architecture. LSTM: long short-term memory.

The architecture of the model used in the study is illustrated in Figure 1. It consists of three LSTM layers along with dropout layers and a fully connected layer at the end. Dropout layers were used to avoid overfitting, which is a typical problem in machine learning tasks. To train this model, we used the TensorFlow package in Python.

## Results

### Observations

**Figure 2:**
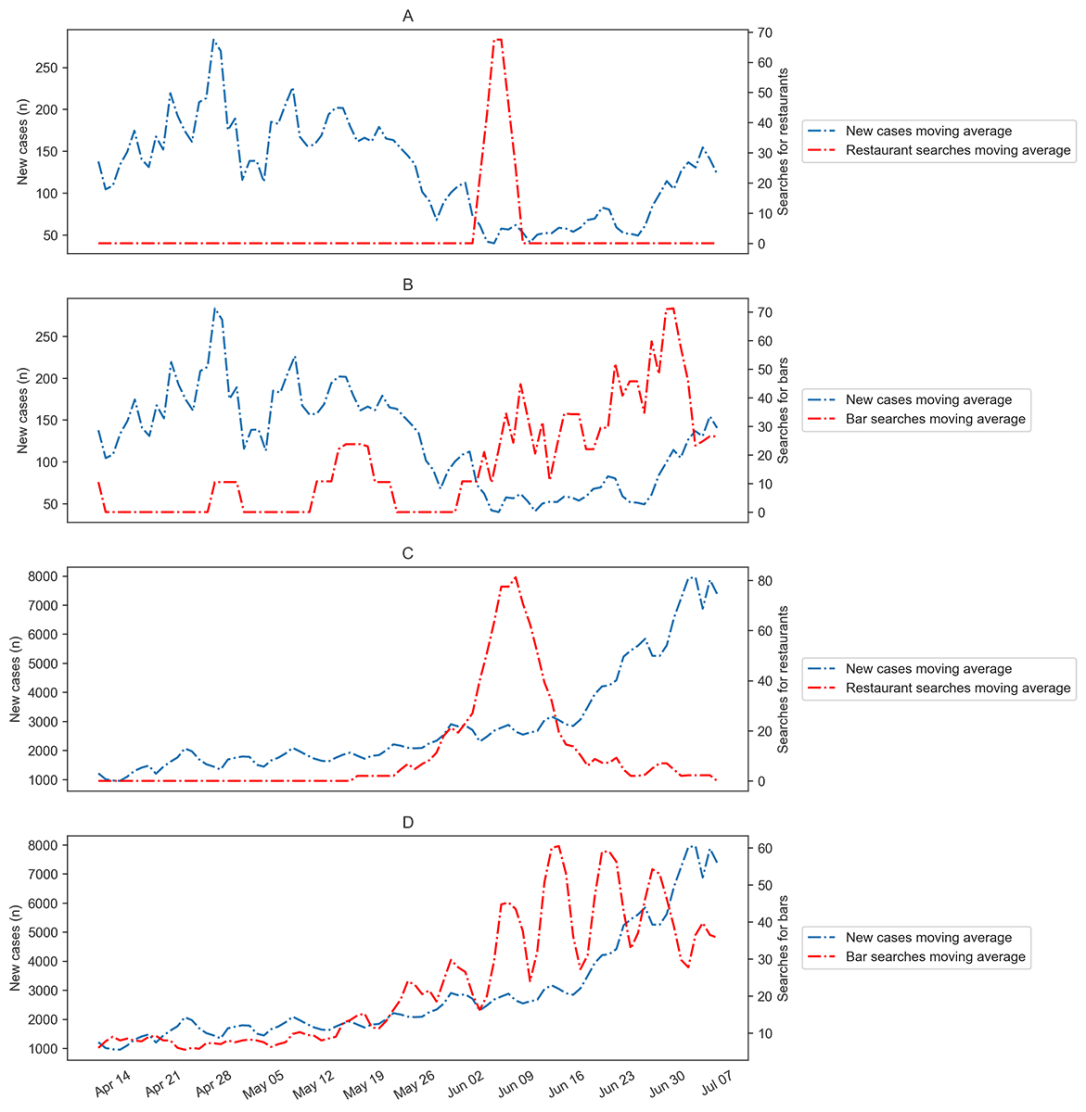
Effects of restaurant and bar search trends on daily cases of COVID-19 in Delaware (A, B) and California (C, D) from April 9 to July 7, 2020.

Investigation of daily new cases and historical trends in search queries related to bars and restaurants showed correlations in some of the states and territories in the United States. For some states and territories, such as California, there was a steep rise in restaurant searches, peaking on June 7. The number of daily new cases showed a drastic increase within 2 weeks of this peak. Considering the bar searches in California, the plot shows an increasing trend, with the peak value appearing on June 13. However, in Delaware, the daily new cases were not profoundly affected by these search trends (Figure 2).

### Granger Causality

In this section, we provide the results of the Granger causality tests for the 10 US states and territories with the highest and lowest numbers of daily new cases as of July 7, 2020.

**Figure 3:**
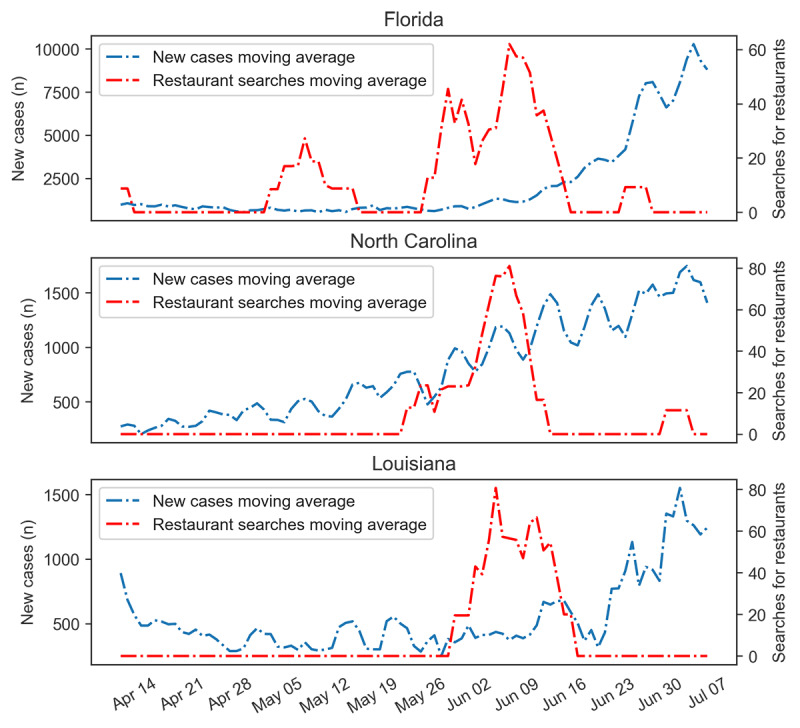
Comparison of restaurant search effects on daily new cases of COVID-19 in Florida, North Carolina, and Louisiana from April 9 to July 7, 2020.

The *P* values for California are small, indicating that the effect of the search queries is significant; hence, these searches can be used to predict daily new cases. Florida and North Carolina are two examples of states in which the effect of restaurant searches is rejected based on the Granger causality test; however, new cases in Louisiana were significantly affected by restaurant searches (Table 1). Figure 3 illustrates the moving average of daily new cases and restaurant search trends for these three states. The high *P* value for Florida is because of the first peak in the restaurant search, which did not change the daily new cases trend. North Carolina has an overall increasing trend; therefore, the effect of the searches was marginal. However, Louisiana was influenced by the sudden changes in restaurant search trends, which affected the number of daily new cases (Figure 3).

**Table 1:** *P* values of the Granger causality tests on daily new cases of COVID-19 for the 10 US states and territories with the most daily new cases from April 9 to July 7, 2020.

Cause → caused	*P* value									
	Texas	Florida	California	Arizona	Georgia	Louisiana	Tennessee	North Carolina	Washington	Pennsylvania
Restaurant searches → new cases	.11	.35	.004	.003	.30	<.001	.09	.53	<.001	.11
Bar searches → new cases	.02	.16	<.001	.04	.001	<.001	.08	.20	.02	.01

Similarly, Table 2 summarizes the *P* values for the Granger causality test for the second group (ie, the 10 states and territories with the fewest daily new cases). Most of the *P* values for these states and territories are not significant.

**Table 2:** *P* values of the Granger causality tests on daily new cases of COVID-19 for the 10 US states and territories with the fewest daily new cases from April 9, 2020, to July 7, 2020.

Cause → caused	*P* value									
	Kansas	Hawaii	New Hampshire	Maine	West Virginia	Rhode Island	Connecticut	Montana	Nebraska	Delaware
Restaurant searches → new cases	.99	<.001	.88	.08	.08	.54	.99	<.001	.99	>.99
Bar searches → new cases	.01	.001	.50	.11	.45	.28	.008	.07	.08	<.001

### Pearson Correlation

In this section, we provide the Pearson correlation results. Tables 3 and 4 summarize these correlations with the corresponding *P* values for each group. Based on these two tables, the linear correlation between the search trends related to bars and restaurants and daily new cases in states and territories with a higher number of daily new cases is more substantial, on average, compared to that for states and territories with fewer daily new cases.

**Table 3:** Pearson correlations between search trends and daily new cases of COVID-19 for the 10 US states and territories with the most daily new cases from April 9 to July 7, 2020.

Variable			Texas		Florida		California		Arizona		Georgia		Louisiana		Tennessee		North Carolina		Washington		Pennsylvania		
**Restaurant searches versus new cases**																							
	Correlation	–0.17		–0.19		0.0		–0.11		–0.2		–0.13		–0.18		0.17		–0.11		–0.23			
	*P* value	.11		.07		.96		.30		.07		.23		.08		.10		.29		.03			
**Bar searches versus new cases**																							
	Correlation	0.11		0.41		0.47		0.31		0.31		0.12		0.39		0.73		0.13		–0.52			
	*P* value	.28		<.001		<.001		.003		.003		.26		<.001		<.001		.20		<.001			

**Table 4:** Pearson correlations between search trends and daily new cases of COVID-19 for the 10 US states and territories with the fewest daily new cases from April 9 to July 07, 2020.

Variable			Kansas		Hawaii		New Hampshire		Maine		West Virginia		Rhode Island		Connecticut		Montana		Nebraska		Delaware
**Restaurant searches versus new cases**																					
	Correlation	–0.05		–0.08		–0.08		–0.08		0.09		–0.08		–0.06		–0.01		–0.05		–0.17	
	*P* value	.62		.43		.45		.42		.35		.42		.55		.85		.61		.10	
**Bar searches versus new cases**																					
	Correlation	–0.20		0.22		–0.11		0.13		0.11		–0.61		–0.22		0.19		0.007		–0.18	
	*P* value	.06		.03		.27		.21		.28		<.001		.04		.07		.94		.09	

### Prediction of New Cases

The prediction results of daily new cases using our deep neural network architecture are provided in this section. The RMSE scores for test data for the US states and territories with the 10 highest and lowest numbers of daily new cases are summarized in Tables 5 and 6 for each model.

**Table 5:** Root mean square error scores for the time series of new COVID-19 cases (baseline), the baseline + restaurant searches time series, and the baseline + bar searches time series for the 10 US states and territories with the most daily new cases from April 9 to July 7, 2020.

Model	Root mean square error									
	Texas	Florida	California	Arizona	Georgia	Louisiana	Tennessee	North Carolina	Washington	Pennsylvania
Baseline	18.00	48.21	24.19	31.35	29.90	39.84	35.88	19.74	26.44	18.70
Baseline + restaurant searches	32.44	43.84	21.86	45.32	33.46	29.36	32.51	22.91	23.92	18.10
Baseline + bars	44.50	32.55	19.89	26.20	36.39	43.51	38.09	26.68	22.75	24.68

**Table 6:** Root mean square error scores for the time series of new COVID-19 cases (baseline), the baseline + restaurants time series, and the baseline + bars time series for the 10 US states and territories with the fewest daily new cases from April 9 to July 7, 2020.

Model	Root mean square error									
	Kansas	Hawaii	New Hampshire	Maine	West Virginia	Rhode Island	Connecticut	Montana	Nebraska	Delaware
Baseline	28.41	51.49	12.09	20.92	26.18	5.37	3.47	29.58	5.49	20.73
Baseline + restaurant searches	25.56	43.64	8.10	14.57	22.55	8.88	3.91	43.34	8.22	20.42
Baseline + bars	34.43	49.01	15.30	21.96	24.15	6.01	4.68	43.27	8.67	12.81

**Figure 4:**
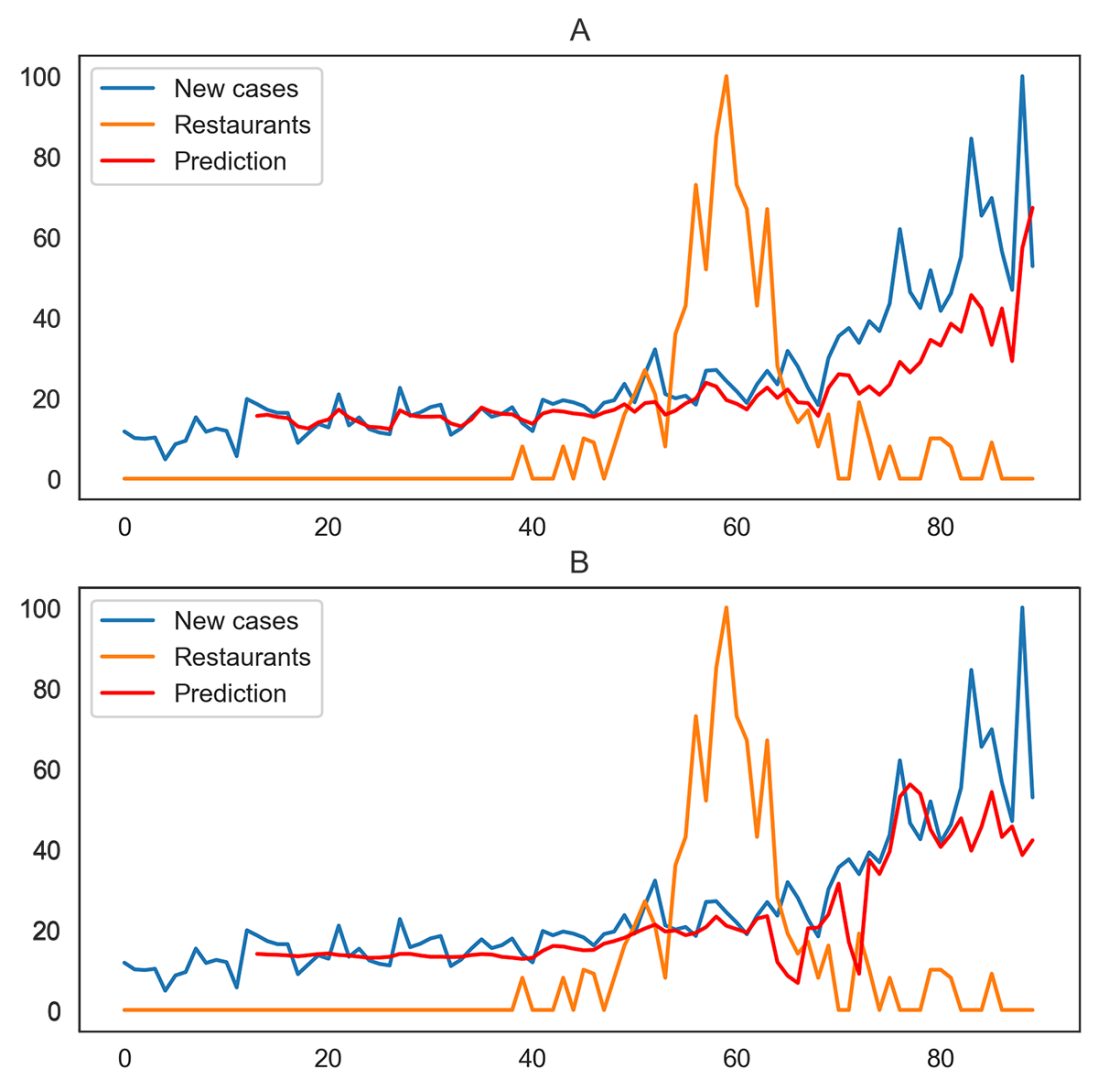
Prediction values for daily new cases of COVID-19 without (A) and with (B) restaurant search trends for California from April 9 to July 7, 2020.

For the states and territories with significant causality effects, the RMSE improves on average. California is an example of a state that shows this improvement (Table 5). Similarly, Figure 4 illustrates the prediction performance with and without considering the restaurant search trends. The predicted values are closer to the actual values when the effect of restaurant searches is taken into consideration in the prediction model.

**Figure 5:**
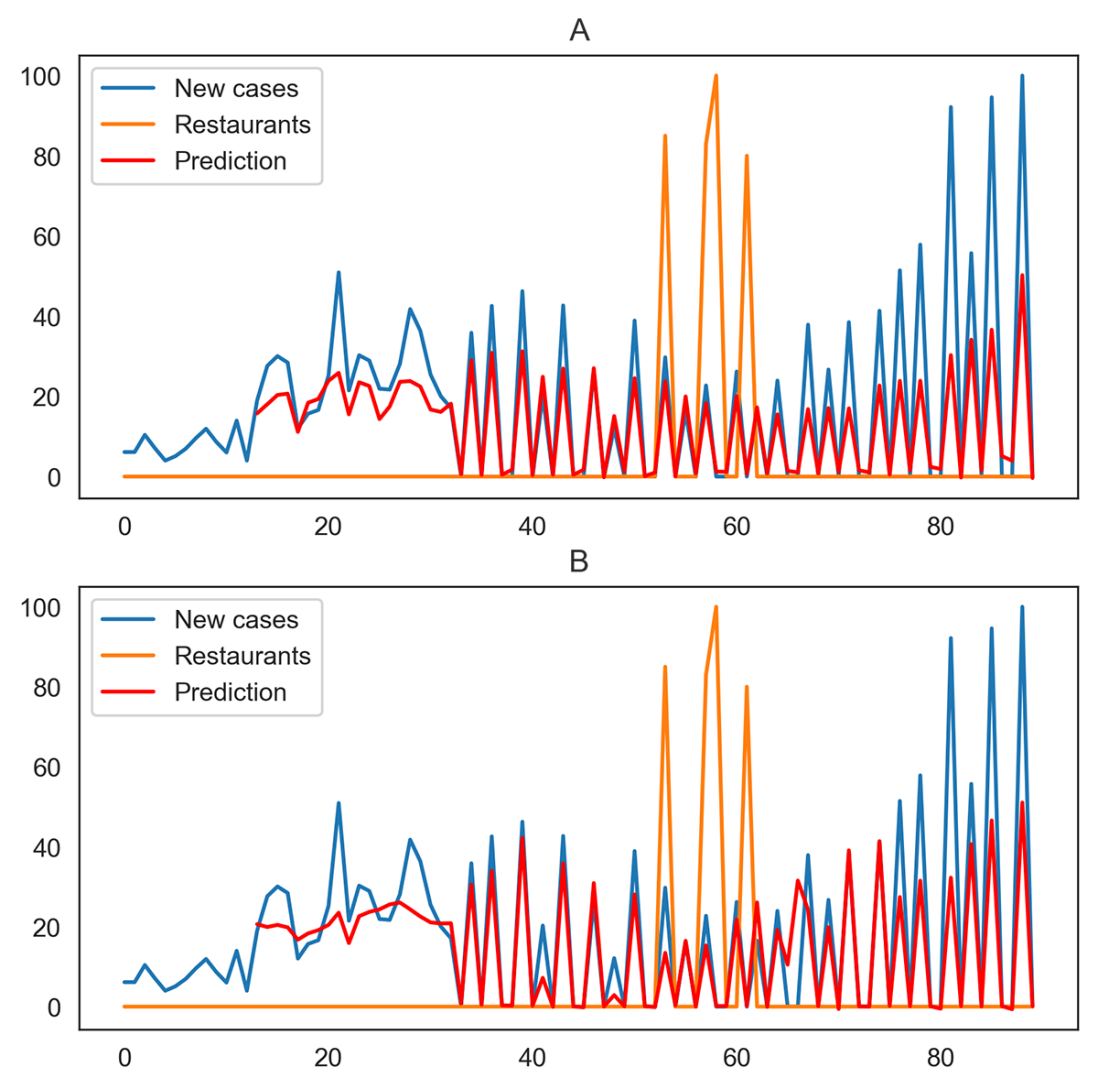
Prediction values for daily new cases of COVID-19 without (A) and with (B) restaurant search trends for Kansas from April 9 to July 7, 2020.

**Figure 6:**
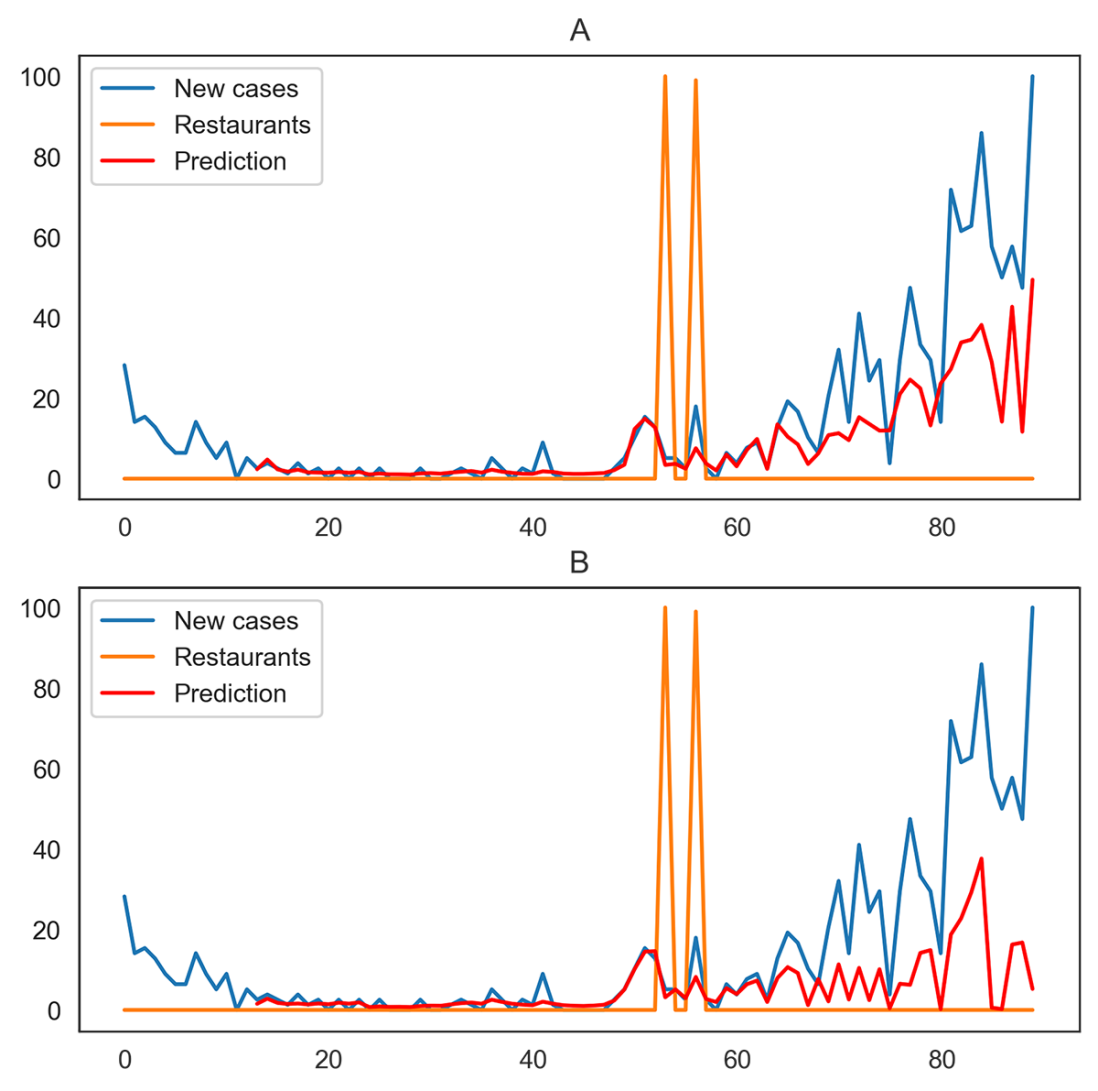
Prediction values for daily new cases of COVID-19 without (A) and with (B) restaurant search trends for Montana from April 9 to July 7, 2020.

For some states, although there was no causality effect for restaurant searches, the RMSE value improved. On the other hand, for states such as Montana, in which the Granger causality test shows a significant effect, the RMSE increased (Table 6). By investigating the time series for these two states (Figures 5 and 6), we can interpret these inconsistencies as arising for two reasons. First, for states such as Kansas, the value improves because of the fluctuation in the new cases time series, which makes the prediction unreliable. Second, as Figures 5 and 6 show, the impulses in restaurant searches for Kansas and Montana are point impulses. These unit jumps cannot significantly improve the prediction of the time series, although they appear in the causality tests.

## Discussion

### Principal Results

To the best of our knowledge, this study is the first analysis that considers the ability of Google search trends related to dine-in restaurants and bars to predict daily new cases of COVID-19 in the United States. Our main findings show that in states and territories with higher numbers of daily cases, the historical trends in search queries related to bars and restaurants (queries related to dine-in venues), which occurred primarily after reopening, significantly correlate with the number of daily new cases on average. In this study, we used statistical methods to validate this effect on the number of daily new cases. One potential reason for this effect could be a smaller population, as this is reflected in the number of daily new cases. The other reason may be the high number of new daily cases, in California for instance, at the time of reopening of restaurants and bars (+2000).

The Granger causality tests show that in some states and territories, the effect of restaurant searches on daily new cases is significant. California is an example of such a state. On May 18, the governor of California announced the easing of criteria for counties to reopen, enabling them to reopen faster than the state, and on May 25, he announced plans for the reopening of in-store shopping [38]. Consequently, there was an increase in restaurant searches, and the peak of the searches occurred on June 7. The number of daily new cases drastically increased within two weeks of the escalation in dine-in restaurant searches. 

A similar trend in bar searches was observed in California. Irrespective of the seasonal effect of the time series, which shows a higher number of searches related to bars during weekends, the average trend in bar searches increased. However, North Carolina was not influenced by restaurant searches. This is because this state showed an increasing average trend irrespective of the other time series. Therefore, the *P* value for the Granger causality is high (.53). In summary, Granger causality showed significant results for states and territories with higher numbers of daily new cases on average.

This study suggests that the effect of restaurant and bar searches is greater in states and territories with higher numbers of daily new cases compared to states and territories that report lower numbers of positive cases every day. On average, in the states and territories with higher numbers of daily new cases, the more significant Granger casualties and higher Pearson correlation values support this fact. Additionally, by taking restaurants and bar searches into account, we can improve the underestimation of the prediction task. We used artificial intelligence models to improve the prediction results of new cases using additional information, namely Google Trends. These Google Trends for searches for restaurants and bars can be useful depending on the time series structure.

According to infodemiology, capturing real-time information and public attitudes can help decision makers to be prepared based on the feedback loop on public data and disease spread [7] and can provide a better estimation of a deadly disease such as COVID-19 in each state to distribute health care–related utilities such as ventilators. In addition, this information can be used to model and analyze food- and lifestyle-related behaviors at the global level based on real-time events [39-41].

### Limitations

There are several limitations to this study. We only used specific search queries for each category. People use different search terms to find the information they are looking for. Moreover, we only considered the effect of restaurants and bar searches on the number of daily cases. Further research could aim to consider the effects of other public places, such as gymnasiums and adventure parks. Another limitation of our study is the limited number of data points for each region (88 samples on average). This limitation, which is a consequence of the daily report data structure, affects the prediction results to a certain degree.

### Conclusions

We investigated the causality effect and correlation of search queries related to dine-in restaurants and bars on the daily numbers of new cases of COVID-19 in the US states and territories with the highest and lowest numbers of daily cases from April 9 to July 7, 2020. We showed that for most of the states and territories with high numbers of daily new cases, the effect of search queries related to bars and restaurants is greater; hence, these searches can be used as additional information for prediction tasks.

## Appendix

Multimedia Appendix 1 P values for the Granger causality tests on daily new cases of COVID-19 for the remaining US states and territories from April 9 to July 07, 2020.

Multimedia Appendix 2 Pearson correlations between search trends and daily new cases of COVID-19 for the remaining US states and territories from April 9 to July 07, 2020.

Multimedia Appendix 3 Root mean square error scores for the new cases time series of COVID-19 (baseline), baseline + restaurant searches time series, and baseline + bar searches time series for the remaining US states and territories from April 9 to July 7, 2020.

Multimedia Appendix 4 P values of the augmented Dickey-Fuller statistics for the stationarity tests for the US states and territories.

## Abbreviations

ADF: augmented Dickey-Fuller
LSTM: long short-term memory
RMSE: root mean square error
VAR: vector autoregression
