# Supplementary material for: The Causality Inference of Public Interest in Restaurants and Bars on Daily COVID-19 Cases in the United States: Google Trends Analysis
Source: JMIR Public Health Surveill. 2021 Apr 6;7(4):e22880. doi: 10.2196/22880 (PMC8025919; doi:10.2196/22880)
Supplement: Multimedia Appendix 1 [file publichealth_v7i4e22880_app1.docx]

**Appendix 1**

Table 7. Granger’s causality test (*P*-values) on daily new cases for the rest of the

states/territories in the US.

| State/Territory | Restaurant search  -> New cases | Bar search -> New Cases |
| --- | --- | --- |
|  |  |  |
| SC |  |  |
|  | .078 | .002 |
| MS |  |  |
|  | .085 | <.001 |
| OH |  |  |
|  | .99 | <.001 |
| AL |  |  |
|  | .003 | .06 |
| NV |  |  |
|  | .11 | <.001 |
| OK |  |  |
|  | <.001 | <.001 |
| MO |  |  |
|  | .12 | .16 |
| VA |  |  |
|  | .89 | .83 |
| MI |  |  |
|  | .77 | .16 |
| NY |  |  |
|  | 1.0 | .75 |
| IL |  |  |
|  | .38 | .91 |
| UT |  |  |
|  | <.001 | .17 |
| MN |  |  |
|  | .002 | .066 |
| WI |  |  |
|  | <.001 | <.001 |
| MD |  |  |
|  | .82 | .69 |
| IA |  |  |
|  | .075 | .001 |
| KY |  |  |
|  | .18 | .47 |
| ID |  |  |
|  | .55 | .013 |
| IN |  |  |
|  | .086 | .31 |
| NJ |  |  |
|  | .96 | .46 |
| AR |  |  |
|  | <.001 | .11 |
| NM |  |  |
|  | <.001 | .33 |
| OR |  |  |
|  | <.001 | .90 |
| MA |  |  |
|  | <.001 | .44 |
| CO |  |  |
|  | .80 | 0.99 |
