# Supplementary material for: The Causality Inference of Public Interest in Restaurants and Bars on Daily COVID-19 Cases in the United States: Google Trends Analysis
Source: JMIR Public Health Surveill. 2021 Apr 6;7(4):e22880. doi: 10.2196/22880 (PMC8025919; doi:10.2196/22880)
Supplement: Multimedia Appendix 2 [file publichealth_v7i4e22880_app2.docx]

**Appendix 2**

Table 8. Pearson correlation between search trends and daily new cases for the rest of the

states/territories in the US.

| State/Territory | Restaurant vs. New cases  (r [P-value]) | Bar vs. New cases (r [P-value]) |
| --- | --- | --- |
|  |  |  |
| SC |  |  |
|  | -0.06 [.52] | 0.48 [<.001] |
| MS |  |  |
|  | -0.12 [.23] | 0.16 [.12] |
| OH |  |  |
|  | -0.21 [.044] | .04 [.69] |
| AL |  |  |
|  | 0.01 [.92] | .41[<.001] |
| NV |  |  |
|  | -0.07 [.45] | 0.42 [<.001] |
| OK |  |  |
|  | -0.11 [.27] | 0.20 [.069] |
| MO |  |  |
|  | -0.08 [.45] | 0.11 [.29] |
| VA |  |  |
|  | 0.16 [.109] | -0.13 [.20] |
| MI |  |  |
|  | -0.44 [<.001] | -0.54 [<.001] |
| NY |  |  |
|  | -0.26 [.012] | -0.47 [<.001] |
| IL |  |  |
|  | -0.19 [.067] | -0.57 [<.001] |
| UT |  |  |
|  | 0.03 [.71] | 0.37 [.002] |
| MN |  |  |
|  | -0.19 [.07] | -0.26 [.010] |
| WI |  |  |
|  | -0.01 [0.89] | 0.20 [.047] |
| MD |  |  |
|  | -0.06 [.51] | -0.53 [<.001] |
| IA |  |  |
|  | -0.14 [.16] | 0.07 [.48] |
| KY |  |  |
|  | -0.10 [.30] | 0.03 [.77] |
| ID |  |  |
|  | 0.0 [.99] | .22 [.03] |
| IN |  |  |
|  | -0.10 [.34] | -0.21 [.043] |
| NJ |  |  |
|  | -0.12 [.23] | -0.47 [<.001] |
| AR |  |  |
|  | 0.12 [.23] | 0.25 [.015] |
| NM |  |  |
|  | 0.04 [.65] | 0.17 [.10] |
| OR |  |  |
|  | -0.04 [.70] | 0.28 [.006] |
| MA |  |  |
|  | -0.06 [.55] | -0.54 [<.001] |
| CO |  |  |
|  | -0.15 [.15] | -0.26 [.011] |
