# Supplementary material for: The Causality Inference of Public Interest in Restaurants and Bars on Daily COVID-19 Cases in the United States: Google Trends Analysis
Source: JMIR Public Health Surveill. 2021 Apr 6;7(4):e22880. doi: 10.2196/22880 (PMC8025919; doi:10.2196/22880)
Supplement: Multimedia Appendix 3 [file publichealth_v7i4e22880_app3.docx]

**Appendix 3**

Table 9. RMSE scores for new cases time series (Baseline), Baseline + Restaurants time series, and Baseline + Bars time series for the rest of states/territories in the US.

| State/Territory | Baseline | Baseline + Restaurants | Baseline + Bars |
| --- | --- | --- | --- |
|  |  |  |  |
| SC |  |  |  |
|  | 27.87 | 55.66 | 55.15 |
| MS |  |  |  |
|  | 27.02 | 24.90 | 27.43 |
| OH |  |  |  |
|  | 22.61 | 23.11 | 27.66 |
| AL |  |  |  |
|  | 28.26 | 46.12 | 41.79 |
| NV |  |  |  |
|  | 26.01 | 35.64 | 32.77 |
| OK |  |  |  |
|  | 19.14 | 36.80 | 38.02 |
| MO |  |  |  |
|  | 34.69 | 31.49 | 35.08 |
| VA |  |  |  |
|  | 8.55 | 10.75 | 54.49 |
| MI |  |  |  |
|  | 15.00 | 15.16 | 18.88 |
| NY |  |  |  |
|  | 3.99 | 2.07 | 19.90 |
| IL |  |  |  |
|  | 6.09 | 4.65 | 25.05 |
| UT |  |  |  |
|  | 25.29 | 29.61 | 49.20 |
| MN |  |  |  |
|  | 19.08 | 17.48 | 28.64 |
| WI |  |  |  |
|  | 38.42 | 28.32 | 32.60 |
| MD |  |  |  |
|  | 5.12 | 12.08 | 13.41 |
| IA |  |  |  |
|  | 27.08 | 21.46 | 19.29 |
| KY |  |  |  |
|  | 19.65 | 18.25 | 23.02 |
| ID |  |  |  |
|  | 50.33 | 44.76 | 52.78 |
| IN |  |  |  |
|  | 18.31 | 22.79 | 12.76 |
| NJ |  |  |  |
|  | 6.09 | 13.53 | 21.76 |
| AR |  |  |  |
|  | 26.70 | 45.51 | 38.47 |
| NM |  |  |  |
|  | 26.02 | 26.07 | 28.85 |
| OR |  |  |  |
|  | 38.59 | 24.31 | 24.92 |
| MA |  |  |  |
|  | 4.60 | 4.30 | 11.57 |
| CO |  |  |  |
|  | 11.01 | 7.03 | 7.39 |
