# Supplementary material for: The Causality Inference of Public Interest in Restaurants and Bars on Daily COVID-19 Cases in the United States: Google Trends Analysis
Source: JMIR Public Health Surveill. 2021 Apr 6;7(4):e22880. doi: 10.2196/22880 (PMC8025919; doi:10.2196/22880)
Supplement: Multimedia Appendix 4 [file publichealth_v7i4e22880_app4.docx]

**Appendix 4**

Table 10. *P*-values of ADF statistics for stationarity test for the states/territories in the US.

| State/Territory | Daily New Cases | Restaurants Trend | Bars Trend |
| --- | --- | --- | --- |
|  |  |  |  |
| TX |  |  |  |
|  | .039 | <.001 | .011 |
| FL |  |  |  |
|  | <.001 | <.001 | <.001 |
| CA |  |  |  |
|  | <.001 | <.001 | .002 |
| AZ |  |  |  |
|  | <.001 | <.001 | <.001 |
| GA |  |  |  |
|  | <.001 | <.001 | <.001 |
| LA |  |  |  |
|  | <.001 | .015 | <.001 |
| TN |  |  |  |
|  | <.001 | <.001 | .001 |
| NC |  |  |  |
|  | <.001 | <.001 | .16 |
| WA |  |  |  |
|  | <.001 | <.001 | <.001 |
| PA |  |  |  |
|  | <.001 | <.001 | <.001 |
| SC |  |  |  |
|  | .25 | <.001 | <.001 |
| MS |  |  |  |
|  | <.001 | <.001 | <.001 |
| OH |  |  |  |
|  | <.001 | <.001 | <.001 |
| AL |  |  |  |
|  | <.001 | <.001 | <.001 |
| NV |  |  |  |
|  | <.001 | <.001 | <.001 |
| OK |  |  |  |
|  | <.001 | <.001 | <.001 |
| MO |  |  |  |
|  | <.001 | <.001 | <.001 |
| VA |  |  |  |
|  | <.001 | <.001 | <.001 |
| MI |  |  |  |
|  | <.001 | <.001 | .002 |
| NY |  |  |  |
|  | <.001 | <.001 | .080 |
| IL |  |  |  |
|  | <.001 | <.001 | <.001 |
| UT |  |  |  |
|  | <.001 | <.001 | <.001 |
| MN |  |  |  |
|  | <.001 | <.001 | <.001 |
| WI |  |  |  |
|  | <.001 | <.001 | <.001 |
| MD |  |  |  |
|  | <.001 | <.001 | <.001 |
| IA |  |  |  |
|  | <.001 | <.001 | <.001 |
| KY |  |  |  |
|  | <.001 | <.001 | <.001 |
| ID |  |  |  |
|  | <.001 | <.001 | <.001 |
| IN |  |  |  |
|  | <.001 | <.001 | <.001 |
| NJ |  |  |  |
|  | <.001 | <.001 | .071 |
| AR |  |  |  |
|  | <.001 | <.001 | <.001 |
| NM |  |  |  |
|  | <.001 | <.001 | <.001 |
| OR |  |  |  |
|  | <.001 | <.001 | <.001 |
| MA |  |  |  |
|  | <.001 | <.001 | <.001 |
| CO |  |  |  |
|  | <.001 | <.001 | <.001 |
| DE |  |  |  |
|  | <.001 | <.001 | <.001 |
| NE |  |  |  |
|  | <.001 | <.001 | <.001 |
| MT |  |  |  |
|  | <.001 | <.001 | <.001 |
| CT |  |  |  |
|  | <.001 | <.001 | <.001 |
| RI |  |  |  |
|  | <.001 | <.001 | <.001 |
| WV |  |  |  |
|  | <.001 | <.001 | <.001 |
| NH |  |  |  |
|  | <.001 | <.001 | <.001 |
| ME |  |  |  |
|  | <.001 | <.001 | <.001 |
| HI |  |  |  |
|  | <.001 | <.001 | <.001 |
| KS |  |  |  |
|  | <.001 | <.001 | <.001 |
